# Transcriptomic profiling of *Poa pratensis* L. under treatment of various phytohormones

**DOI:** 10.1038/s41597-024-03119-0

**Published:** 2024-03-15

**Authors:** Chen Meng, Xiaomei Peng, Yu Zhang, García-Caparrós Pedro, Yumeng Li, Yanni Zhang, Yuanwen Duan, Xudong Sun

**Affiliations:** 1 National Engineering Laboratory for Resource Development of Endangered Crude Drugs in Northwest China, Key Laboratory of Medicinal Resources and Natural Pharmaceutical Chemistry, Ministry of Education and School of Life Sciences, Shaanxi Normal University, Xi’an, 710119 PR China; 2 The Germplasm Bank of Wild Species, Yunnan Key Laboratory of Crop Wild Relatives Omics, Kunming Institute of Botany, Chinese Academy of Sciences, Kunming, 650201 Yunnan China; 3 University of Chinese Academy of Sciences, Beijing, China; 4 Department of Agronomy, University of Almeria, 04120 Almeria, Spain

**Keywords:** Plant sciences, Plant hormones

## Abstract

*Poa pratensis* L. (Poaceae) is a valuable grass across the north hemisphere, inhabiting diverse environments with wide altitudinal span, where ubiquitous various kinds of stresses. Phytohormones would be helpful to improve tolerance to abiotic and biotic stresses, but the responses of transcriptome regulation of *P. pratensis* to exogenous phytohormones application remain unclear. In this study, we explored the alteration of plant physiological responses by the application of phytohormones. Aiming to achieve this knowledge, we got full-length transcriptome data 42.76 Gb, of which 74.9% of transcripts were completed. Then used 27 samples representing four treatments conducted at two time points (1 h and 6 h after application) to generate RNA-seq data. 371 and 907 common DEGs were identified in response to four phytohormones application, respectively, these DEGs were involved in “plant hormone signal transduction”, “carbon metabolism” and “plant-pathogen interaction”. Finally, *P. pratensis* basic research can gain valuable information regarding the responses to exogenous application of phytohormones in physiological indicators and transcriptional regulations in order to facilitate the development of new cultivars.

## Background & Summary

*Poa pratensis* L. is a valuable forage and turf grass widely inhabiting temperate for its high adaptability^1^, including infrequent mowing, trampling tolerance, and versatility^2^. However, susceptibility to diseases and pests, sensitivity to heat, drought and salt of this species limit the wide cultivation^3,4^.

The exogenous application of phytohormones can lead to the accumulation of endogenous phytohormones and improve plant defense^5^. Therefore application of exogenous phytohormones would be of great help in improving tolerance to abiotic and biotic stresses^6,7^. For instance, salinity and low temperatures adversely affect the ability of plants to respond to abscisic acid (ABA)^8^. In the context of abiotic stresses, indole-3-acetic acid (IAA) assumed a prominent role in regulating plant growth^9^. Similarly, jasmonic acid (JA) was recognized as a significant factor in plant adaptation to both abiotic and biotic stresses^10^. These phytohormones treatments alter some typical plant physiological indicators, such as relative water content (RWC), contents of chlorophyll, stomatal conductance (Gs) and so on^11,12^. Physiological indicators of plants are a good way to detect whether a plant is suffering from biotic or abiotic stresses^13^. The utilization of transcriptomics studies enables the examination of the transcriptional regulation of all genes within cells and tissues. Transcriptomics analysis has been used for molecular mechanisms for coping with biotic and abiotic stresses in recent years^14^. Currently, the only existing transcriptome data of *P. pratensis* pertains to osmotic stress condition^15^, salt stress condition^16^ and powdery mildew infection^17^. However, it is generally unclear how *P. pratensis* responds to exogenous phytohormones in physiological indicators and transcriptional regulations, making it difficult to further utilize this forage^18^.

The purpose of the present study was to analyze the transcriptional change of *P. pratensis* exerted by four different phytohormones, including 6-Benzylaminopurine (6-BA), ABA, IAA, and JA. The analyses were presented at two different time points at the same stage of the plant. Physiological analysis revealed that exogenous application of phytohormones such as 6-BA, IAA, ABA, and JA altered physiological parameters which may have increased the ability of plants to cope with stresses. Samples were processed for full-length transcriptome sequencing, generating 42.76 Gb of PacBio sequencing data, BUSCO was applied to assess the completeness of the transcriptome after de-redundancy. The transcriptome of *P. pratensis* was assessed with quality control. 27 RNA-seq libraries from *P. pratensis* treated with different phytohormones were constructed. Using the results of principal component analysis (PCA) to detect differences in gene expression level between samples. A total of 371 and 907 common differentially expressed genes (DEGs) were identified through 1 h and 6 h, respectively. By Gene Ontology (GO) and Kyoto Encyclopedia of Genes and Genomes (KEGG) analysis, the most enriched GO entries were “binding”, “cell part” and “metabolic process”, and these DEGs were found to be mainly involved in “plant hormone signal transduction”, “carbon metabolism” and “plant-pathogen interaction”. As a result of the extensive physiological indicators and transcriptome data obtained, future studies of phytohormones for plant tolerance in *P. pratensis* are likely to provide valuable information for the creation of high-quality cultivars.

## Methods

### Plant growth and exogenous phytohormones treatments

**Fig. 1:**
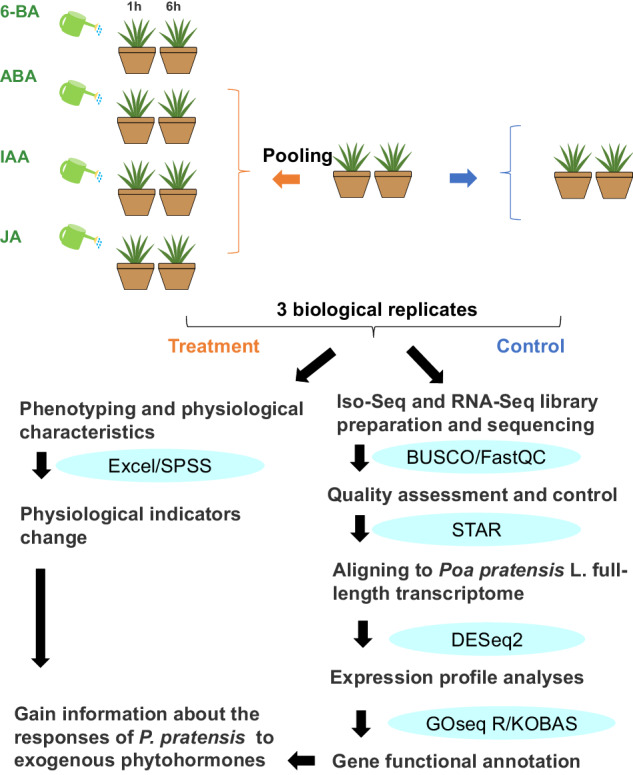
Flowchart of experimental design of this study. To capture the maximal alteration in the transcriptome of *P. pratensis* plants, the 6-BA, ABA, IAA, and JA treatments were applied. Transcriptome alterations were found by comparing responses between treated plants and their equivalent controls. Briefly, 14-d-old plants were supplied with CK, 6-BA, ABA, IAA and JA treatments, in parallel, three biological replicates per condition were used for transcriptome sequencing. All raw reads were quality-controlled before aligning to the *P. pratensis* reference genome.

In the Spring of 2022, seedy seeds were sterilized and placed in petri dishes for 14 days (growth conditions: temperature 23 °C, relative humidity 65–75%, photoperiod 12 h, light intensity 50 μmol m^−2^s ^−1^). The plants were subjected to exogenous phytohormones treatments: 6-BA, ABA, IAA and JA for 1 h and 6 h. Control plants were treated with distilled water (CK). Fully developed leaves were collected in both control and treated plants in the different harvesting times point (0 h in control and 1 h and 6 h after the application). For physiological determinations and RNA extraction, each sample was separated into two parts. We froze a portion of the RNA extraction in liquid nitrogen within 30 minutes and stored it at −80 °C for later use. Chemical agents were listed in detail along with their final concentrations in Table 1. An overview of the workflows for analysis of exogenous phytohormones treatment and transcriptome data was provided (Fig. 1).

**Table 1:** The information on exogenous phytohormones.

The name of chemical agents	Abbreviation	Specie of phytohormone	Final concentration
**6-Benzylaminopurine**	6-BA	Cytokinin	10 μM
**(+)-Abscisic Acid**	ABA	Abscisic acid	0.2 μM
**Indole-3-acetic acid**	IAA	Auxin	2 μM
**(±)-Jasmonic acid**	JA	Jasmonic acid	10 μM

### Plant physiological characteristics

The leaf RWC of *P. pratensis* was calculated according to the methodology reported by Slatyer in 1967^19^ to determine the RWC of *P. pratensis*. The extraction of chlorophyll was carried out using the mixed acetone-ethanol precipitation method^20^. The absorbances of the photosynthetic pigments were determined spectrophotometrically, at wavelengths of 665 nm, 649 nm and 470 nm. The contents of chlorophyll a, chlorophyll b and carotenoids were calculated using the formula of Holm (1954) and Wetstein (1957) (mg g^−1^ fw)^21^. At 9:00–11:00 am, on a sunny day, five healthy and fully developed leaves were selected and the Gs was measured using the SC-1 Leaf Poromete (Decagon Devices, Inc., https://www.decagon.com)^22^.

### RNA extraction

27 samples of fully developed leaves were taken from each of the four phytohormones treatments in the two times points as well as from plants without phytohormones applied (CK), Leaf samples were used for total RNA extraction, and the Eastep Super Total RNA Extraction (Promega Co., Ltd., Shanghai, China) was used for analysis. The extracted total RNA was subjected to quality testing. RNA purity was assessed using NanoDrop ND-1000 (Thermo Fisher Scientific, Waltham, MA, USA). RNA quality was assessed using the RNA Nano 6000 Assay Kit of Agilent Bioanalyzer 2100 (Agilent Technologies, Santa Clara, CA, USA).

### PacBio Iso-Seq and RNA-Seq library preparation and sequencing

For next-generation sequencing (NGS) transcriptome sequencing, RNA samples of acceptable quality were used to construct sequencing libraries with the TruSeq RNA library Prep Kit (Illumina, CA, USA), and the qualified libraries were sequenced on Illumina sequencing platform at Biomarker Technologies (Beijing, China). For full-length transcriptome sequencing. By Synthesis Single Molecule Real-time (SMRT) technology, RNA samples of acceptable quality were used to construct sequencing libraries with the SMARTer PCR cDNA Synthesis Kit (Clontech, Mountain View, CA, USA), and the qualified libraries were processed for full-length transcriptome sequencing on the PacBio sequencing platform at Biomarker Technologies (Beijing, China).

### Iso-Seq and RNA-Seq data processing

Processing of raw data of full-length transcriptome was conducted by SMRTlink 5.1 software (https://www.pacb.com/videos/tutorial-minor-variant-analysis-smrt-link-v5-0-0/). LoRDEC software was used to correct additional nucleotide errors in consensus reads based on Illumina RNA-seq data. Low-quality concordant sequences obtained from individual samples were corrected with corresponding Illumina RNA-seq data using proovread^23^ software to improve full-length transcriptome sequencing accuracy. Then we use CD-HIT (version 4.6.1)^24^ combine reads of full-length transcriptome with high similarity and remove the redundancy. Full-length transcriptome completeness was assessed using the BUSCO (version 3.0.2) pipeline^25^. The raw reads of NGS transcriptome from the machine were quality controlled using FastQC (https://www.bioinformatics.babraham.ac.uk/projects/fastqc/fastqc_v0.12.1.zip) and filtered for low quality data. The calculations were carried out simultaneously for Q20, Q30, GC-content, and clean data sequence duplication levels.

### Analysis of differential gene expression and quantification of gene expression levels

The full-length transcriptome sequencing data in the study were used as the reference transcriptome. Gene expression level were quantified by Kallisto(version 0.46.1)^26^ based on transcripts generated from circular consensus (CCS) data. To eliminates the affect of gene length and data size on gene expression level, Fragments Per Kilobase of transcript per Million mapped reads (FPKM) was applied here as a standard method to estimate gene expression level. The box plot presented the dispersion of gene expression within a sample and the comparison of overall expression among samples^27^. FPKM of different samples were then subjected to PCA using TBtools (version 2.042)^28^. FPKM were used to estimate gene expression levels. The differential expression analysis of two conditions using the DESeq 2(version 1.6.3) (https://github.com/mikelove/DESeq2) was performed for all samples with biological replicates. Using Benjamini and Hochberg’s false discovery rate control method, *P* values were adjusted. DEGs were searched for using the DESeq 2 (FDR < 0.01 and a Fold Change ≥ 2). The results of the DEG analysis were also represented by heatmaps, Venn diagrams and Volcano plot. Heatmaps were constructed using R/pheatmap(version 1.0.2) for gene visualization. Venn diagram by Venny (version 2.1.0) (https://bioinfogp.cnb.csic.es/tools/venny/index.html). Volcano plot generated by the ViDGER(version 1.22.0) (https://bioconductor.org/packages/release/bioc/html/vidger.html) using a DESeq 2 data set.

### Gene functional annotation

Sequences of non-redundant transcripts were annotated by DIAMOND against databases including NR^29^, Swissprot^30^, GO^31^, COG^32^, KOG^33^, Pfam^34^, KEGG^35^.

GO Orthology^31^ of transcripts was obtained by the underlying software InterProScan (version 5.34–73.0)basic on the InterPro database. The GOseq R (version 2.18.0) packages implemented GO enrichment analysis based on Wallenius non-central hypergeometric distributions, which can compensate for the gene length bias in DEGs^36^.

KEGG Orthology^35^ of transcripts was obtained by the above processes. As a method of testing whether KEGG pathways were enriched statistically by differential expression genes, we used KOBAS^37^ software.

Subsequently, the obtained analytical results of GO and KEGG were enriched was produced using R/clusterProfiler (version 3.10.1)^38^.

### Statistical analysis

The experiment was conducted with at least three biological replicates for each treatment in a completely randomized design. Data were classified using Microsoft Excel (2019) and analyzed by ANOVA using SPSS Statistics (version 20.0). The two-tailed paired Student’s t test was used to calculate statistically significant differences in gene expression. A *P* value ≤ 0.05 was considered statistically significant.

## Data Records

The raw data were submitted to the National Genomics Data Center (NGDC) Genome Sequence Archive (GSA) with full-length transcriptome data (Pacbio sequencing) accession number CRA006867^39^, and NGS transcriptome data (Illumina sequencing) accession number CRA006889^40^.

Supplementary materials are available on the Figshare data management platform^41^. It provides transcript sequences; offers function annotation of this transcriptomic; furnishes count data of heat map and venny original data, physiological and biochemical analysis count data DEG analysis and other original data; and contains outputs of GO and KEGG analyses.

## Technical Validation

### Effects of exogenous phytohormones treatments on physiological characteristics

Plant leaves are sensitive to external changes, and under different constraint environments leaves are able to change their own structure and morphology to cope with the stresses. To investigate the physiological effects of exogenous phytohormones application on *P. pratensis*, we examined important physiological indicators of plant leaves.

**Fig. 2:**
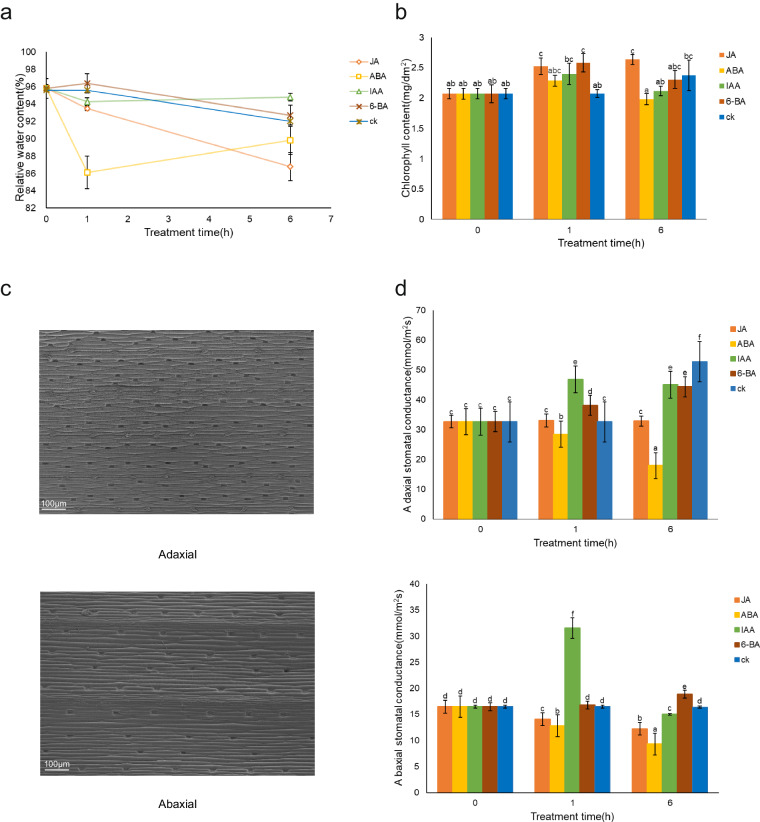
Effects of exogenous phytohormones treatments on physiological characteristics. (**a**) Comparison of RWC of plants under different treatments. (**b**) Comparison of chlorophyll content of plants under different treatments. (**c**) Morphology of *P. pratensis* leaf blades under swept electron microscopy. (**d**) Comparison of changes in Gs of plants under different treatments.

Leaf water content of leaves is positively correlated with stress tolerance^42^. The Leaf RWC of *P. pratensis* decreased gradually over time, and after the application of exogenous phytohormones. After following IAA treatment, the leaf RWC the decrease from 95.79% to 86.12%, then increase to 89.84%, while the same expression pattern was found in ABA and it’s RWC was 94.78%, which was highest at 6 h treatment (*P* < 0.05). The results showed a clear effect of phytohormones treatments on the regulation of the leaf RWC (Fig. 2a).

Abiotic stresses inhibit plant photosynthesis, affect chloroplast stability and induce chloroplast degradation^43^. After the four phytohormone treatments, the chlorophyll content was significantly increased only in plants to which JA was applied, increase with time from 2.07 mg/dm^2^ to 2.53 mg/dm^2^ and then to 2.64 mg/dm^2^, other phytohormone treatments result in a rapid short-term increase and then decrease in chlorophyll content due to stress (*P* < 0.05). Chlorophyll content directly affects the strength of photosynthesis, and chloroplast degradation in turn triggers early plant senescence, which is detrimental to plant participation in the response to adverse conditions (Fig. 2b).

Stomata are important sites for gas and water exchange in plants, and changes in plant stomata affect the ability of plants to adapt to the external environment^44^. There were differences in stomatal density between adaxial and abaxial surfaces in *P. pratensis* (Fig. 2c). The statistical results revealed that the Gs was greater in the adaxial surface than in the abaxial surface with a difference of 8.64–36.37 mmol/m^2^s. The light energy intercepted by the adaxial or the abaxial surfaces was not equal, resulting in the instability of the Gs difference (*P* < 0.05). In adaxial surface, the Gs of the plants non-treated with phytohormones became higher over time, but the Gs of the plants became lower after the application of ABA, decrease with time from 32.63 mmol/m^2^s to 28.43 mmol/m^2^s and then to 17.93 mmol/m^2^s, (*P* < 0.05). We observed the same phenomenon in the abaxial surface, but the change in Gs in the abaxial surface of non-treated plants was not significant(*P* < 0.05) (Fig. 2d). At the same time, we observed that after the application of IAA, *P. pratensis* showed a dramatic increase from 16.47 mmol/m^2^s to 31.53 mmol/m^2^s in Gs at 1 h, after which it decreased to 15 mmol/m^2^s *(P* < 0.05). These results showed that some phytohormones treatments can alter Gs traits, which can increase plant stress tolerance.

The results showed that exogenous phytohormones application enhanced plant resistance to adversity stress by examining the physiological indicators of *P. pratensis*. Specific changes need to be judged based on the results of subsequent transcriptome sequencing data.

### Quality assessment

**Fig. 3:**
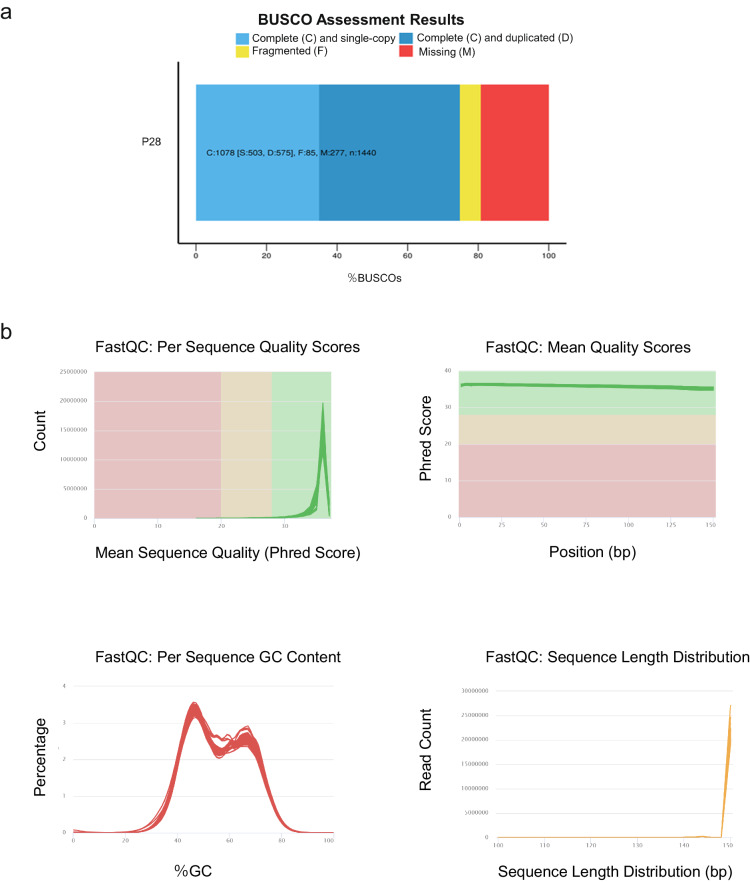
Results of raw read preprocessing. (**a**) Assessment results of transcriptome completeness. (**b**) Per sequence quality scores, mean quality scores, per sequence GC scores and sequence length distribution.

A total of 398,247 CCS reads were produced, in which 320,071 were identified as full length non-chimeric (FLNC) reads. FLNC reads were clustered into 141,574 consensus sequences. A total of 141,548 high quality sequences were obtained by polishing on consensus sequences. Generating 42.76 Gb of PacBio sequencing data. After removing redundant reads, 97,549 non-redundant FLNC reads were obtained. By using BUSCO 2.3, CD-HIT transcripts were assessed for completeness. Based on the results, 74.9% of transcripts were complete, with single-copy BUSCOs accounting for 34.9% and duplicate BUSCOs accounting for 40.0%. Our database contained only 85 fragmented BUSCOs and 277 missing BUSCOs out of 1,440 BUSCO groups searched (Fig. 3a). As a result of these findings, we were able to confirm that our database was complete and was available for further research.

Our 27 polymerase reads were achieved on Illumina HiSeq The sequencing joints and primers in polymerase reads were intercepted and then filtered into low-quality data (Table 2). Using clean data as an example, we have shown the quality control results (Fig. 3b). The result showed that Mean Quality Scores and Per Sequence Quality Scores of the sequencing results were of excellent quality. GC content was consistent at 54–57% and more than 85% of both sequencing reads reached Q30.

**Table 2:** Sample sequencing data evaluation statistics.

Samples	Read Number	Base Number	GC Content	% ≥ Q30
**P-6BA-1-1**	21,450,667	6,422,094,754	55.08	94.43
**P-6BA-1-2**	20,628,547	6,172,877,210	55.70	94.78
**P-6BA-1-3**	20,898,797	6,257,354,848	55.47	93.62
**P-6BA-6-1**	20,070,135	6,009,507,768	55.58	94.33
**P-6BA-6-2**	25,343,719	7,587,326,730	55.54	92.48
**P-6BA-6-3**	22,411,827	6,711,229,914	54.31	92.52
**P-AB-1-1**	21,839,469	6,541,034,472	55.30	93.97
**P-ABA-1-2**	20,647,087	6,182,987,136	55.57	93.14
**P-ABA-1-3**	23,049,835	6,900,821,056	55.69	93.98
**P-ABA-6-1**	21,041,618	6,291,239,422	55.02	94.63
**P-ABA-6-2**	21,140,228	6,327,847,164	55.11	94.02
**P-ABA-6-3**	22,277,578	6,673,404,358	55.71	93.63
**P-IAA-1-1**	22,673,037	6,790,020,024	55.19	93.46
**P-IAA-1-2**	27,912,173	8,352,046,332	55.30	93.10
**P-IAA-1-3**	22,485,524	6,735,385,860	56.07	94.79
**P-IAA-6-1**	22,564,518	6,757,421,452	55.53	94.25
**P-IAA-6-2**	23,972,703	7,180,725,820	54.85	94.41
**P-IAA-6-3**	20,009,460	5,992,576,902	54.89	93.78
**P-JA-1-1**	19,872,473	5,952,013,346	54.99	92.43
**P-JA-1-2**	21,612,515	6,473,416,762	54.78	92.65
**P-JA-1-3**	22,885,578	6,853,425,662	54.97	92.79
**P-JA-6-1**	19,947,830	5,974,416,774	54.74	92.71
**P-JA-6-2**	25,059,575	7,505,315,960	54.82	92.35
**P-JA-6-3**	20,198,172	6,049,512,206	54.93	93.50
**Pp-ck-1**	19,434,546	5,819,411,358	55.28	94.61
**Pp-ck-2**	22,109,619	6,621,831,080	56.07	94.27
**Pp-ck-3**	19,654,688	5,884,329,672	55.05	94.04

Read Number: Total number of pair-end Reads in Clean Dat; Base Number: Clean Data the total number of bases; GC Content: Clean Data GC content, the percentage of both G and C bases in Clean Data to the total bases; % ≥ Q30: Clean Data Percentage of bases with mass values ≥ 30.

**Fig. 4:**
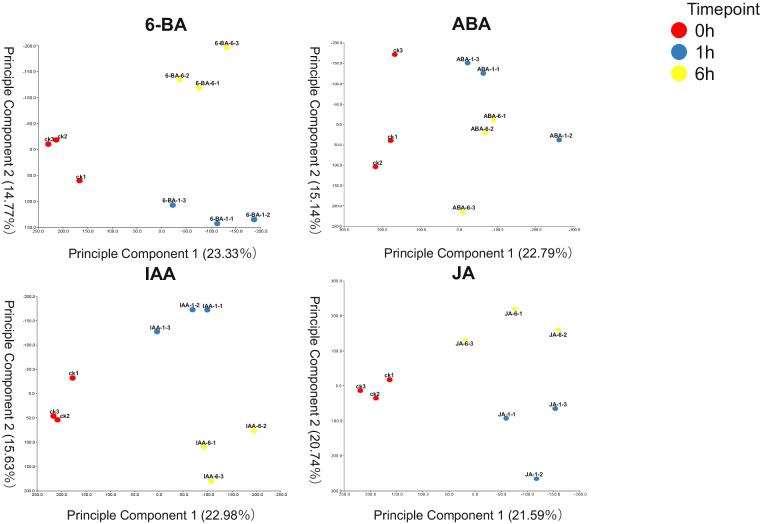
Principal components analysis for each stress. Represented the PCA of 6-BA, ABA, IAA, and JA, compared with control check treatment.

Gene expression level was quantified based on the full-length transcript. To eliminate the effect of gene length and data size on gene expression level, FPKM was applied here as a standard method to estimate gene expression level. The PCA model was used to check the quality and natural variations among different treatments. After PCA analysis was performed on the FPKM of 27 samples representing the different treatments and time points tested, PC1 showed 21.59–22.33% values while PC2 showed 14.77–20.74% values (Fig. 4).

### Differential expression analysis

**Fig. 5:**
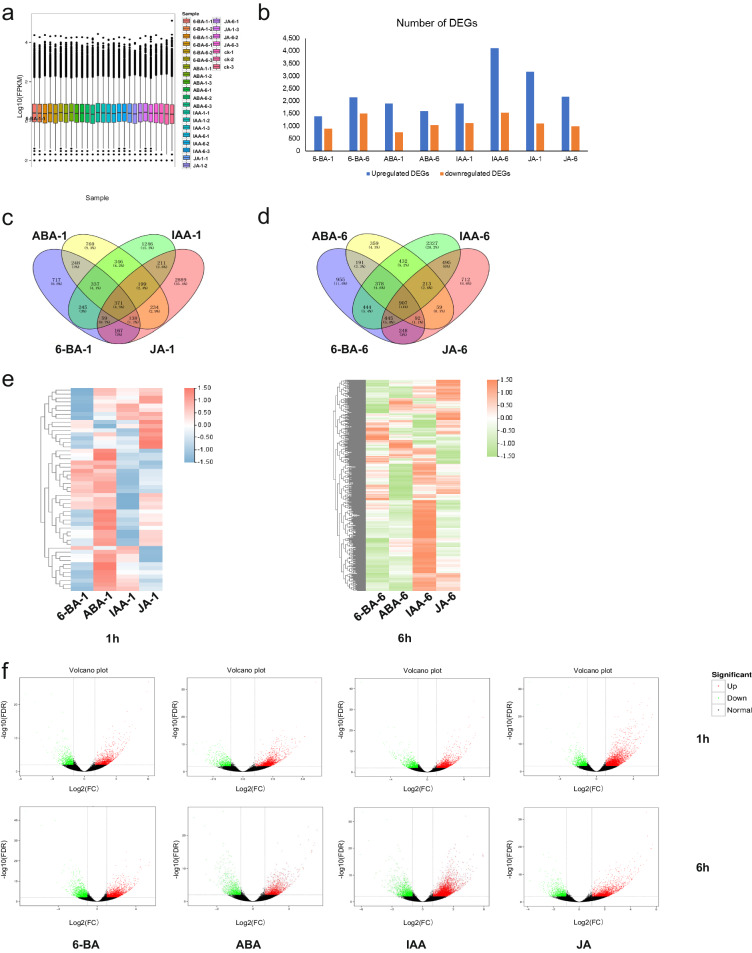
Global assessments of transcriptome data. (**a**) The overall expression level of each sample via the dispersion of gene expression in each sample. (**b**) The number of DEGs in *P. pratensis* under exogenous phytohormones treatments. The DEGs were determined with |log2 fold change (FC)| ≥ 0.5, and an adjusted *P* value < 0.05. (**c**-**d**) Venn diagram depicted the number and overlap DEGs from different phytohormone treatments. (**e**) Hierarchical clustering of differentially expressed. The expression level of genes [Log2(FPKM + 1)] was presented as different colors based on the scale bar. (**f**) Volcano Plot of differentially expressed transcripts. Upregulated DEGs were highlighted in red, downregulated DEGs showed in green, whereas non-differentially DEGs showed in black, with an adjusted *P* value < 0.05 and FC > |2|.

The mapped reads were converted into read counts for each *P. pratensis* gene in order to quantify global gene expression patterns for multiple biotic and abiotic stresses. In a boxplot, the normalized read count distributions of all samples are shown (Fig. 5a). Differential expression analysis between sample groups was performed with DESeq 2, obtaining a set of differentially expressed transcripts between nine biological conditions. Fold Change ≥ 2 and FDR < 0.01 were used as screening criteria. The number of DEGs for 8 treatments were compared with the control (Fig. 5b). Among them, JA had the largest number of DEGs in 1 h, and IAA had the largest number of DEGs in 6 h.

**Fig. 6:**
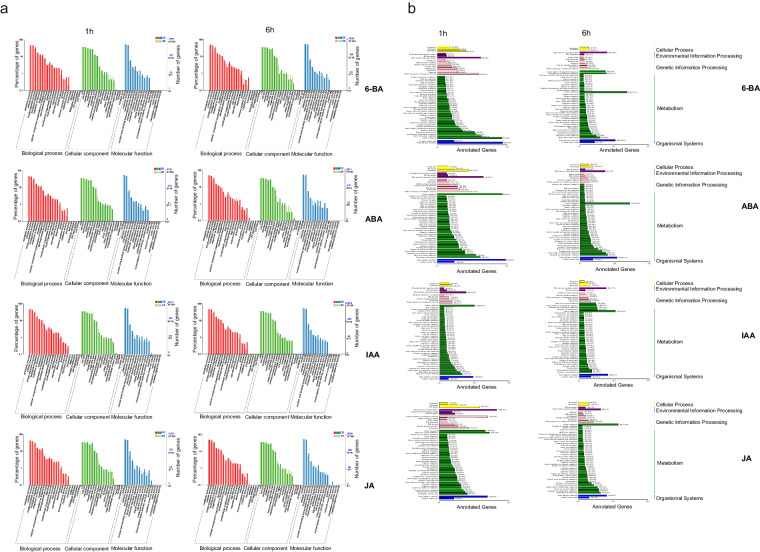
Function analysis. (**a**) The enrichment of DEGs to specific GO terms. The terms with two bars significantly different from each other can be picked up as potential targets for further analysis of functions. (**b**) Classification of DEGs KEGG annotation.

In addition, we have analyzed the DEGs of different phytohormones treatments in 1 h (Figs 5c and 6h (Fig. 5d). The results showed the regulation of the four phytohormone interactions where the time change resulted in a change in the number of DEGs between phytohormones treatments from 8504 to 7350, with 371 and 907 overlapping DEGs identified, respectively. In 1 h, among the 371 DEGs, ABA exhibited a different expression pattern than other phytohormones, with more up-regulated DEGs. In 6 h, among the 907 DEGs, IAA exhibited a different expression pattern than other phytohormones, with more up-regulated DEGs (Fig. 5e). At the beginning of the treatments, the plants were more sensitive to the application of ABA and IAA treatments, while at the end of the treatments, the plants were more sensitive to IAA and JA treatments. The Volcano plots showed that the expression pattern was consistent across all samples (Fig. 5f).

### Functional annotation of transcripts

The DEGs of *P. pratensis* that deal with different phytohormones at different times were analyzed using GO enrichment analysis. According to the results, all DEGs were classified into three categories, which included “molecular functions”, “cellular components”, and “biological processes”. The 30 most enriched GO entries were selected based on the corrected *P* value. In the molecular functions, the main terms were “binding” and “catalytic activity”, in the cell components were “cell part” and “cell”, and in the biological processes was “metabolic process” (Fig. 6a). All these transcripts are important resources for genetic manipulations of *P. pratensis* in the future.

The KEGG annotation classification revealed that annotated sequences represented mostly “plant hormone signal transduction”, “carbon metabolism” and “plant-pathogen interaction” in all samples (Fig. 6b). The great potential possessed by plant hormone signaling networks could enable plants to rapidly alter their metabolic mechanisms in response to biotic and abiotic stresses^45^. This suggests that the increase in plant resistance due to the application of exogenous phytohormones may be through the regulation of plant hormone signal transduction, carbon metabolism and plant-pathogen interaction. Differently, the results showed that the “starch and sucrose metabolism” pathway was only enriched in *P. pratensis* treated by JA for 1 h and the expression of DEGs such as hexokinase (HXK), invertase (INV), glutamine synthetase (GS), sucrose synthase (SS), ADP-glucose pyrophosphorylase (AGPase) and β-amylase in “starch and sucrose metabolism” pathway of *P. pratensis* treated by JA for 1 h were closely related to biotic stresses^17^, revealing a new possible way for JA to cope with stresses. Enrichment factors and fisher test were applied in the determination of enrichment degree and significancy of the pathway. Enrichment of DEGs in KEGG pathways showed same results of KEGG annotation classification, the result can available on the Figshare data management platform^46^.

The distinctive patterns of gene expression and GO and KEGG enrichments suggest that these data will help to determine the interaction between different phytohormones at different times, disentangling the evolutionary mechanisms of adaptation to unfavorable environments. At the same time, helps to speculate on the pathways of phytohormones in stress resistance, which will lay the foundation for subsequent research on forage resource improvement and germplasm.

## Data Availability

The analysis results of this research were performed using the BMKCloud platform (www.biocloud.net). We followed all published bioinformatics tool manuals and protocols for the execution of all software and pipelines. The software version and code/parameters have been described in the Methods section. There was no custom code used during this study for the curation and/or validation of the dataset.
